# Metascape provides a biologist-oriented resource for the analysis of systems-level datasets

**DOI:** 10.1038/s41467-019-09234-6

**Published:** 2019-04-03

**Authors:** Yingyao Zhou, Bin Zhou, Lars Pache, Max Chang, Alireza Hadj Khodabakhshi, Olga Tanaseichuk, Christopher Benner, Sumit K. Chanda

**Affiliations:** 1 Genomics Institute of the Novartis Research Foundation, 10675 John Jay Hopkins Drive, San Diego, CA 92121 USA; 2 Immunity and Pathogenesis Program, Infectious and Inflammatory Disease Center, Sanford Burnham Prebys Medical Discovery Institute, 10901 North Torrey Pines Road, La Jolla, CA 92037 USA; 3 Department of Medicine, University of California, San Diego, 9500 Gilman Drive, La Jolla, CA 92093 USA

## Abstract

A critical component in the interpretation of systems-level studies is the inference of enriched biological pathways and protein complexes contained within OMICs datasets. Successful analysis requires the integration of a broad set of current biological databases and the application of a robust analytical pipeline to produce readily interpretable results. Metascape is a web-based portal designed to provide a comprehensive gene list annotation and analysis resource for experimental biologists. In terms of design features, Metascape combines functional enrichment, interactome analysis, gene annotation, and membership search to leverage over 40 independent knowledgebases within one integrated portal. Additionally, it facilitates comparative analyses of datasets across multiple independent and orthogonal experiments. Metascape provides a significantly simplified user experience through a one-click Express Analysis interface to generate interpretable outputs. Taken together, Metascape is an effective and efficient tool for experimental biologists to comprehensively analyze and interpret OMICs-based studies in the big data era.

## Abstract (web summary)

With the increasing obtainability of multi-OMICs data comes the need for easy to use data analysis tools. Here, the authors introduce Metascape, a biologist-oriented portal that provides a gene list annotation, enrichment and interactome resource and enables integrated analysis of multi-OMICs datasets.

## Introduction

It has become a standard practice to employ one or more OMICs assays to deconstruct the molecular mechanisms underlying a biological system. Since each study ultimately results in a list containing dozens or hundreds of gene candidates, it is essential to leverage existing biological knowledge through understanding the representation of known pathways or complexes within these datasets. Providing this molecular context can facilitate the interpretation of systems-level data and enable new discoveries. Common queries include: What pathways or biochemical complexes are enriched?^1^; What are the functional roles of identified protein complexes?^2^; Which candidate proteins are secreted, contain a transmembrane domain, or are otherwise druggable?^3^; Or are there any chemical probes available for a rapid candidate validation^4^ ? Critically, when multiple gene lists are analyzed, either from common or orthogonal platforms, the identification of consistent underlying pathways or networks can help decipher authentic signals above the experimental noise^5^. Conversely, pathways selectively enriched in specific lists can help elucidate critical molecular features that distinguish experimental conditions. Thus, gene-list analysis web portals have become a necessity for modern biological research. For example, DAVID^6^, a popular web-based annotation tool has been employed by more than 4000 published studies per year since 2014, underscoring the importance of OMICs analysis engines in biological research.

To guide the development of a next-generation tool, we studied 25 existing data analysis portals^7,8^, focusing on database updates, coverage of analyses, coherence of interfaces, and interpretability of outputs. While each platform maintains strong feature sets (Supplementary Data 1), multiple portals are required to accomplish a complete systems-level analysis workflow, leading to a fragmented user experience. For example, a user analyzing proteomics data may need to use one tool to convert protein identifiers into gene symbols, a second tool to perform pathway enrichment analysis, a third tool to assess protein interaction networks, followed by other tools to produce high-quality visualizations of the data. Users are not only required to learn the details behind how to use each interface successfully, but they must also learn how to integrate the outputs from multiple types of analyses and file formats in order to generate useful results. For the inexperienced user, this can pose a significant barrier to entry. Thus, it is likely that significant levels of existing biological knowledge are often inadvertently omitted during the analysis of OMICs datasets due to the disintegrated nature of data sources and analysis tools. Ideally, a broad range of biological relationships and classifications can be assessed within one integrated portal.

Additionally, the increased accessibility of systems-level technology platforms has promoted experimental strategies that rely on orthogonal OMICs approaches, including transcriptome analysis, genetic screens, and proteomics, to interrogate an experimental system. This approach enables a more comprehensive assessment of the molecular features of a biological process and reduces false positive/negative activities associated with individual platforms^9^. Thus, meta-analysis of multiple gene lists to identify commonly-enriched and selectively-enriched pathways is likely to become an essential component of large-scale data analysis. Methods for the analysis of multiple gene lists continue to be an area of active investigation, which highlights the necessity to develop analytical strategies that extend beyond the consolidation and analysis of individual lists, and implement approaches that will enable emergent insights that cannot be extracted using current approaches^10,11^. Our survey found that multi-gene-list meta-analysis is a feature often missing from existing tools (Supplementary Data 1).

In addition to a combination of diverse knowledgebases and the synthesis of analysis results across related gene lists, we propose that biologist-oriented portals would need to incorporate additional critical features, including future-proofing, usability, and interpretability, to maximize their utility. Specifically, analysis portals must continually be kept up to date, since using outdated databases can severely impact analysis and interpretation of OMICs data. It has been estimated that 67% of ~3900 publications in 2015 missed 74% of potential new biological insights as a consequence of the use of obsolete database content in many analysis portals^7^. This remains a concern to date, as the databases underpinning the interpretation of a majority of published studies are over 2 years old^12^.

Additionally, a well-designed interface that requires a minimal number of user interactions (clicks) reduces the barrier to entry for new users, or those with limited computational training. Nevertheless, simplification must be balanced with providing users sufficient information to understand and, if required, adjust statistical and computational criteria within the analysis workflow. This feature would provide expanded access to data analysis tools and sources for experimentalists that have limited experience with OMICs analysis, while enabling more advanced users to adjust analytical parameters to customize analyses.

Furthermore, it is essential that analysis results are readily decipherable, highlighting the key, non-redundant results that will be critical for informing future studies. Platforms that go beyond exporting tabular annotation information to produce graphical summaries of results tend to enhance the global interpretation of complex OMICs datasets. The resulting output analyses should also adopt presentation formats that facilitate data dissemination among scientists.

With these design criteria in mind, we developed Metascape to harness the best practices of OMICs data analyses that have emerged over the last decade into one integrated portal. Here, we describe the features of this platform, which provide enhanced rendition of systems-level data, designed to promote the development of actionable hypotheses. These include data-engineering solutions for over 40 knowledgebases, as well as integrated and simplified workflows for gene annotation, membership search, and multi-list comparative analysis. Furthermore, we describe unique graphical outputs that enhance reporting and boost data comprehension. Taken together, Metascape provides an integrated and user-friendly web tool designed to facilitate multi-platform OMICs data analysis and interpretation for the experimental research community.

## Results

### User experience overview

To illustrate the features and capabilities of Metascape, we utilized three previously published influenza genetic screens^13–15^ as examples in this study (see Methods). These studies utilize genome-wide RNAi screening in cells to identify host factors that modulate influenza replication rates. Specifically, single-gene-list analyses were performed with gene targets identified in the Brass et al. study^13^, and multi-gene-list analyses were performed with the combination of Brass et al., Karlas et al^14^., and Konig et al.^15^ target lists. The step-by-step illustration of the Metascape analysis interface for both single- and multiple-list input scenarios are available in Supplementary Figures 1–7. While these examples showcase the analysis of RNAi functional genomics studies, Metascape is equally capable of analyzing lists of genes from multiple assay types, including transcriptomics, epigenetics, proteomics, and others.

**Fig. 1:**
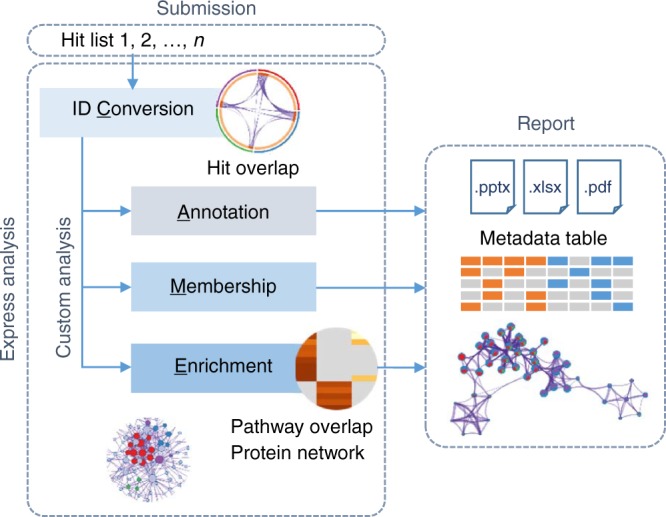
Schematic outline of the Metascape analysis workflow. Upon gene list submission, Custom Analysis enables users to navigate the analysis workflow and output a report. The Express Analysis instead takes a streamlined approach by running the analysis steps with popular default settings, simplifying the user experience

The primary Metascape user experience is provided through a one-click analysis interface (Express Analysis, Supplementary Fig. 1). The user first provides a single or multiple input gene lists, then launches an automated analysis workflow consisting of four major components: identifier conversion, gene annotation, membership search, and enrichment analysis (CAME). An analysis report summarizing key results is produced, accompanied by an Excel workbook, a PowerPoint presentation, and a Zip package containing all supporting data files (Fig. 1).

The Metascape interface was designed to limit user operations to a minimum number of interactions. Input data is automatically processed to recognize identifier types, such as Entrez Gene ID, gene symbol, RefSeq number, Ensembl ID, etc., without user specification. The file input supports multiple flexible formats (Supplementary Figure 1) and can incorporate an optional background gene list to customize the enrichment analysis. Gene identifiers from ten popular model organisms are recognized by Metascape (see Methods), and automatic orthologue mapping enables gene lists to be analyzed in the context of species-specific databases across any of these ten organisms. The target analysis species is human by default, since human-centric databases are often the most comprehensive. Importantly, we have observed that 63% of Metascape analyses to date utilize human datasets.

The concept behind Express Analysis is to encapsulate the most widely used analysis options and best analysis practices into a streamlined approach. For example, Express Analysis automatically includes gene annotation metadata that are likely to provide valuable context for candidate interpretation in most cases, such as their brief description, key biological processes involved, protein functions, subcellular locations, and roles in canonical pathways (Supplementary Figure 3). It incorporates a core set of default ontologies for enrichment analysis, including GO processes^16^, KEGG pathways^17^, Reactome gene sets^18^, canonical pathways^19^, and CORUM complexes^20^ (Supplementary Figure 5). Express Analysis also automatically sets statistical filters to remove ontology terms and interactome networks that do not meet minimal statistical requirements (Supplementary Figure 5). As an alternative to the Express Analysis, advanced users can control the four individual steps of the CAME workflow and overwrite default settings through the Custom Analysis workflow (Fig. 1, Supplementary Figures 2–6).

### Identifier conversion analysis

The first step of the Metascape analysis workflow is gene identifier conversion (Supplementary Figure 2). Metascape automatically recognizes the popular gene identifier types described above, as well as primary locus names from various model organism databases, such as SGD^21^, FlyBase^22^, WormBase^23^, etc. Source identifiers are mapped onto a unique Entrez Gene ID list prior to analyses, as a significant number of bioinformatics knowledgebases underlying Metascape rely on Entrez Gene IDs as their primary keys. Deprecated Entrez Gene IDs are also recognized and replaced by successor IDs when encountered.

Many gene annotation, pathway, and protein interaction databases are primarily compiled for human genes/proteins. For instance, the size of the mouse interactome encompasses only ~6% of the available human interactome^24^, even though many of these interactions are likely conserved across species. Therefore, it can be beneficial to cast gene candidates obtained in model organisms into their human orthologs prior to analysis. Ortholog mapping is a built-in feature and can be triggered by explicitly choosing the target analysis species (Supplementary Fig. 1), which will instruct subsequent analysis steps to use resources for that particular species.

### Gene annotation

Detailed annotations for genes provide biological context to serve as important selection criteria when choosing candidates for follow-up studies. Metascape integrates annotation information from over 40 knowledgebases (Supplementary Figure 8, Supplementary Data 2), encompassing gene descriptions, gene summaries, disease implications, genomic variants, subcellular localizations, tissue expression, the availability of chemical probes, etc. Express Analysis delivers nine descriptive fields by default, however, as illustrated in Supplementary Figure 3, the Custom Analysis feature allows the user to append up to 47 columns of metadata to a gene annotation spreadsheet.

To assimilate this level of information using current tools, the experimentalist is required to first identify the appropriate knowledgebases that contain the required information. Since many of these portals do not incorporate gene list upload features for batch annotation, users must, in certain cases, search individual gene candidates one at a time. For example, it becomes an onerous undertaking to identify all extracellular proteins through UniProt^25^, or all chemical probes through DrugBank^26^, using a list with hundreds of gene candidates. Thus, Metascape’s automated gene annotation feature provides users with detailed annotation information not otherwise easily accessible, aiding in data interpretation of large-scale datasets and providing prioritization criteria for candidate genes.

### Membership search

The membership search feature in the Custom Analysis workflow allows users to apply specific query keywords, such as “infection” or “kinase”, against knowledgebase category term names and description fields, then review and select those matched terms that most accurately describe the process of interest (Supplementary Figure 4). Metascape will then dynamically construct a column of binary flags indicating whether each gene in the input gene list is a member of the matched ontology terms. This allows the user to identify genes with specific functions or features, and reduces the reliance on existing hierarchical ontologies during data interpretation. For example, Metascape offers two approaches to identify transmembrane proteins candidates. First, there are pre-integrated transmembrane predictions provided by Ensembl^27^ or UniProt that are extractable through the gene annotation feature as described above. An alternative is to use the Membership tools to search the keyword “transmembrane” within the GO Cellular Component catalog to identify member genes. Although these two approaches are complementary in this specific example, membership search becomes critical when metadata is not readily available through pre-integrated data sources. For instance, highlighting known infection-related genes for an influenza study cannot be accomplished via standard gene annotation. Despite the fact that this knowledge is embedded in the ontology databases, it has not been explicitly compiled in most annotation resources and, thus, is not readily accessible to experimentalists. The binary result data from membership searches can also be visualized within nested pie charts (Supplementary Figure 9).

### Enrichment clustering

**Fig. 2:**
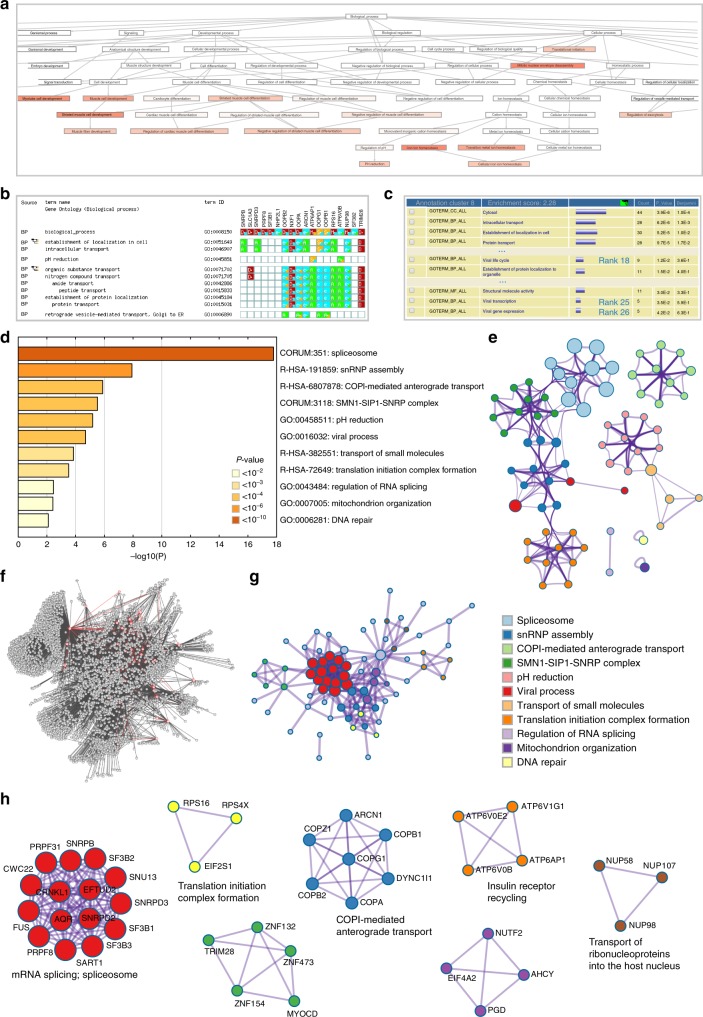
Visualizations of functional enrichment and interactome analysis results. **a** Screenshot of a portion of the directed acyclic graph rendered by Babelomics^53^ based on enriched GO terms. **b** Screenshot of a portion of the gene-term association matrix rendered by g:profile^42^, where enriched GO terms are organized hierarchically. **c** Screenshot of a portion of the tabular display of enriched terms rendered by DAVID based on all GO terms, KEGG pathways, Reactome, and CORUM. The terms related to “viral gene expression” were missed in the visualizations generated by Babelomics and g:profile, but were identified by DAVID (in cluster 8 with ranks around 20). **d** Metascape bar graph for viewing top non-redundant enrichment clusters, one per cluster, using a discrete color scale to represent statistical significance. **e** Metascape enrichment network visualization showing the intra-cluster and inter-cluster similarities of enriched terms, up to ten terms per cluster. Cluster annotations are shown in color code. **f** A complex interactome network generated by g:profile with the Brass gene list. **g** Metascape visualization of the interactome network formed by all 121 gene candidates from the Brass list, where the MCODE complexes are colored according to their identities. **h** Seven MCODE complexes automatically identified in Metascape, colored by their identities. Their functional labels are generated based on the top-three functional enriched terms, if available

Enrichment analysis comprises the core of most existing gene annotation portals. During enrichment analysis, the input gene list is compared to thousands of gene sets defined by their involvement in specific biological processes, protein localization, enzymatic function, pathway membership, or other features. Gene sets whose members are significantly overrepresented in the input gene list are reported to users to serve as putative biological insights into their study. A neglected problem in most current analyses is that redundancies in descriptors and ontologies can often complicate interpretation of the output. For example, ontology terms found in GO form a hierarchical structure of increasing granularity, making the terms inherently redundant. Terms across different ontology sources, such as GO, KEGG, and MSigDB^19^, etc. can be closely related as well. As a result, functional enrichment analysis can identify overlapping or related terms, making it difficult to extract non-redundant and representative processes to report in the analysis output. This problem is demonstrated in examples from other analysis portals using the Brass list (Fig. 2a, b), which report the enrichment of dozens of highly-related categories. During post-processing of data generated through the Metascape analysis, Kappa similarities^28^ among all pairs of enriched terms are computed and used to first hierarchically cluster terms into a tree then cast subtrees into clusters of similar terms (see Methods). By absorbing most redundancies into representative clusters, enrichment clustering eliminates confounding data interpretation issues that can arise from the reporting of multiple ontologies.

In comparison with Fig. 2a–c, d shows the Metascape enrichment analysis results of the Brass list, where bars are discretely colored to encode *p*-values of increasing statistical significance. The bar graph representation nevertheless does not capture inter-cluster similarities and intra-cluster redundancies. To address this, we have implemented a visualization approach called enrichment networks. As shown in Fig. 2e, enrichment networks are created by representing each enriched term as a node and connecting pairs of nodes with Kappa similarities above 0.3, forming a network that can be depicted using Cytoscape^29^. Nodes can be colored to reflect either their statistical *p*-values or cluster memberships. Redundant terms within a cluster naturally drive the formation of tight local complexes due to their high intra-cluster similarities, while clusters are occasionally bridged through terms with similarities reflecting the relatedness of two separate processes.

Although Metascape introduces the above features to facilitate the interpretation of enrichment analysis results, it is not designed to supersede existing functional enrichment analysis portals. For users solely conducting enrichment analysis for a single gene-list, some existing tools, such as Enrichr^30^ and ToppFunc^31^, contain additional gene signature collections and they remain a powerful approach to enrichment analysis.

### Protein network analysis

Analyzing gene lists in the context of protein interactions can help illuminate biochemical complexes or signal transduction components that govern biological outputs^32^. However, a typical gene list of a few hundred human proteins can form highly complicated network layouts, making interpretation difficult. This is readily exemplified by the network output of other analysis portals using the Brass list (Fig. 2f). These are colloquially referred to as hairball networks. Although such large networks are generally statistically significant, they do not yield directly actionable insights unless the experimentalist has specific expectations of the data. To infer more biologically interpretable results, Metascape applies a mature complex identification algorithm called MCODE^33^ to automatically extract protein complexes embedded in the large network. Taking advantage of Metascape’s functional enrichment analysis capability, the three most significantly enriched ontology terms are combined to annotate putative biological roles for each MCODE complex. As shown in the example, Metascape results are relatively simple to visualize and interpret (Fig. 2g, h).

### Multi-gene-list meta-analysis

The proliferation and general adaptation of orthogonal OMICs platforms, including proteomics, genomics, functional genetic screens, and metabolomics, has made the meta-analysis of multiple gene lists a critical and largely unaddressed need. Routine comparative approaches include the use of Venn diagrams to identify hits that are common or unique to certain gene lists. We, and others, have previously reported that an overlap between OMICs datasets is more readily apparent at the level of pathways or protein complexes^9,34,35^, which can be considerably more challenging to implement without extensive computational expertise.

Metascape has been designed to allow cross comparison of an arbitrary number of gene lists across both gene identities and ontologies. Specifically, upon the submission of multiple gene lists, all candidates are merged into one list, while encoding the original lists as additional binary membership columns (Supplementary Fig. 10). This pivoted layout greatly simplifies the representation of analysis results, as both gene annotation and membership search can be applied to the combined list the same way as in a single gene list input. Using a single output spreadsheet to incorporate evidence collected from each gene list thus enables efficient global gene filtering and prioritization. The overlap among the gene lists is visualized using a Circos plot^36^ (Supplementary Fig. 11), which is a more intuitive and scalable representation compared to a Venn diagram.

**Fig. 3:**
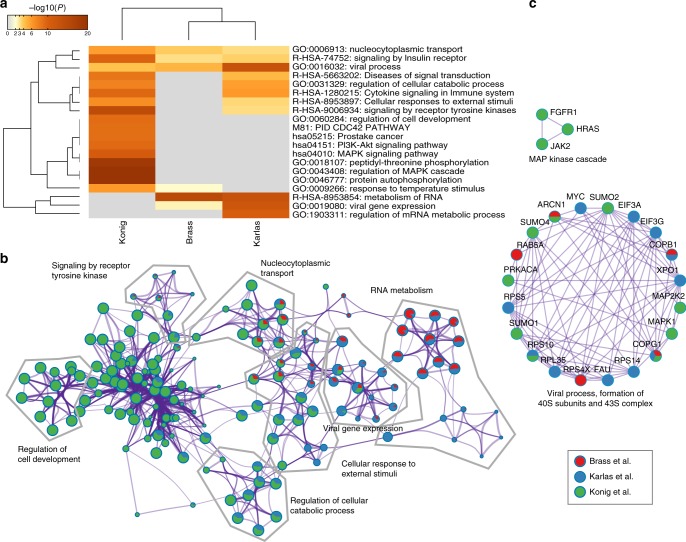
Visualizations of meta-analysis results based on multiple gene lists. **a** Heatmap showing the top enrichment clusters, one row per cluster, using a discrete color scale to represent statistical significance. Gray color indicates a lack of significance. The category GO:0016032 (viral process) is common to all studies, while GO:0046777 (protein autophosphorylation) is enriched exclusively in a single study, and is therefore likely a process associated with one particular experimental system. **b** Enrichment network visualization for results from the three gene lists, where nodes are represented by pie charts indicating their associations with each input study. Cluster labels were added manually. Color code represents the identities of gene lists. The network shows that processes such as viral gene expression, nucleocytoplasmic transport, and cellular response to external stimuli are generally shared among all three lists. RNA metabolism is shared between the Brass and Karlas lists; cellular development processes are mostly shared between the Karlas and Konig lists. **c** Selected MCODE components identified from the combined list of 541 genes, where each node represents a protein with a pie chart encoding its origin. The complex related to viral process is shared among all three lists, while the complex related to the MAP kinase cascade is specific to the Konig list. Supplementary Figure 12 presents all MCODE components identified

To facilitate the understanding of pathways (and pathway clusters) that are shared between, or selectively ascribed to, specific gene lists, additional visualizations were developed for Metascape. First, Metascape depicts top enriched clusters and their enrichment patterns across multiple gene lists as a clustered heatmap (Fig. 3a). The heatmap is complemented by an enrichment network where each network node is represented by a pie chart, where the sector size is proportional to the number of genes originated from each gene list (Fig. 3b). This representation is designed to be a biologically intuitive illustration of selective or shared pathway clusters. Importantly, this approach can also be applied to visualize protein–protein interaction networks to enable the elucidation of common/selective complex components enriched across OMICs datasets (Fig. 3c, Supplementary Fig. 12).

### Maintaining updated data sources

Metascape integrates over 40 knowledgebases (Supplementary Fig. 8) to support comprehensive analyses over ten highly studied organisms. Over time, some underlying data sources may adopt new access mechanisms, rename file packages, or be impacted by modifications to cross-referenced databases. Even if each data source is 99% robust, a system relying on 40 independent sources can only be 67% reliable. As the result of this compound effect, unexpected changes are encountered during data synchronization processes at frequencies significant enough to hinder attempts to automate content updates, which eventually results in outdated portals. DAVID^6^, a heavily utilized annotation tool accounting for over 80% of all functional enrichment portal usage, was not updated between 2010 and 2016, and then again not since 2016^12^. Our latest analyses on DAVID indicated 4–10% of the input gene candidates (varied by identifier types) are not recognized. Further analysis of the human GO archive data indicated 44% of annotation records have been added or modified since its last update. This is in agreement with a semantic similarity measure suggesting that 20% of genes change their functional identity over a two-year period^37^. Along with other outdated portals, it was estimated that thousands of studies were affected, and the potential impact on follow-up studies could last for years to come^7^.

**Fig. 4:**
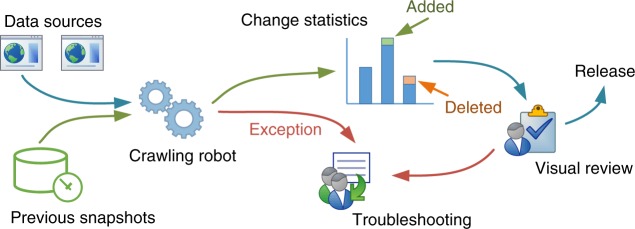
Schematic diagram of the semi-automatic data synchronization workflow

Metascape has adopted a novel two-phase approach to overcome this critical issue and ensure monthly updates (Fig. 4). In the first phase, individual data sources are automatically crawled, wrangled, and assembled according to a predefined topological order of dependency, where gene identifier resources are processed before dependent annotation resources. When significant changes or unexpected errors occur while processing a data source, a database’s last known compatible copy from a previous update is reused, and an internal alert is issued to Metascape quality control (QC) developers (see below). Using this strategy, conflicts in any given database component will not stop the timely completion of the Metascape update cycle. It is important to note that more subtle inconsistencies in data content can be harder to detect and may not trigger these exceptions. For example, the accidental deletion of records for a certain model organism in NCBI^38^ may go undetected. The Metascape pipeline automatically carries out a detailed comparison between the current database and the new updated database, summarizing count differences into a bar graph web report, indicating the number of data records that are added or removed as illustrated in Fig. 4. In the second phase, a Metascape QC member inspects the graphical report for suspicious changes in record counts. If no concerning issues are identified, the release candidate is approved, and the database update goes to production. Alerts received during the whole process are manually examined and addressed in parallel. Taken together, this workflow ensures that Metascape users are not adversely impacted by outdated data sources.

### Usage and citation analysis

Metascape has been in development since 2014, and a beta version of the software was initially released in late 2015. The portal usage data, as well as feedback from users, have been decisive in correcting or refining underlying concepts that drove the architectural design and features of Metascape. Some usage statistics are particularly enlightening. Among all recently recorded analysis sessions, ~78% users chose Express Analysis over Custom Analysis, implying that Express Analysis successfully captures a set of core algorithms and settings suitable for the majority of analysis needs. Six percent of analyses contained more than one gene list, indicating a demand for meta-analysis in the current research landscape. Data collected on input identifier types indicates gene symbol and synonyms are the most widely used (77%), followed by Ensembl identifiers (11%), Entrez Gene ID (4%), and RefSeq (3%). The remaining IDs are mostly UniProt IDs and model-organism-specific IDs. Microarray IDs are hardly used (<0.2%), likely reflecting the shift of the gene expression technology platform from microarrays to next-generation sequencing. About 63% of analyses were performed using human identifiers, followed by 31% using mouse. Other model organisms such as *D. rerio, R. norvegicus, A. thalianai*, etc. accounted for the remainder. Interestingly, 3% usage took advantage of the ortholog mapping feature to mostly cast mouse/rat gene lists into human context for analysis. These data validate a need to support multiple gene/protein identifiers and multiple model organisms.

**Fig. 5:**
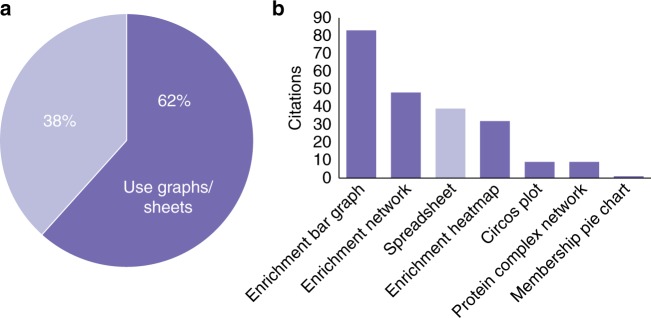
Citation statistics illustrating the use of Metascape visualizations in publications. **a** 62% of publications adopted visualizations prepared by Metascape. **b** Visualization types sorted according to their popularity, where the enrichment bar graph, network, and heatmap are frequently used to summarize functional enrichment outputs

At the time of writing, our citation survey using Google Scholar identified 62% of the citations have adopted Metascape graphical outputs directly in publications (Supplementary Data 5), underscoring the utility of tools that generate high-quality data visualizations (Fig. 5a). The top three most used visualizations are the enrichment bar graph, enrichment network, and enrichment heatmap, highlighting the utility of the tool for functional enrichment analyses (Fig. 5b). In addition to graphical outputs, Metascape analysis generates two Excel spreadsheets providing gene annotation and functional enrichment results (Supplementary Data 3, 4, Supplementary Figure 7). These data outputs have been directly used in over 16% of publications as supplementary materials to support analysis conclusions (Supplementary Data 5). Since Circos plots and enrichment heatmaps are two visualization tools specifically designed for multiple gene lists only, and a bar graph is only used for a single gene list, we estimated that 70% of the published studies studied a single gene list and 30% studied multiple gene lists. This finding, together with our web portal usage data, highlight the increasing usage of multi-OMICs approaches in current studies, and suggest that meta-analysis has become an important tool for the analysis of OMICs datasets.

## Discussion

The systematic categorization of genes by their functions by the Gene Ontology Consortium (GO) has initiated the widespread practice of functional enrichment analysis; many portals have been developed over the years and have led to the continual refinement of gene list analysis algorithms^39–41^. Metascape was not developed to replace existing specialized tools, but to provide a pipeline to assimilate many of the powerful features of these analysis platforms to enable integrative, end-to-end analysis of OMICs-level datasets.

Our study of 25 existing analysis portals found that the underlying databases were often out-of-date (Supplementary Data 1). Although portals such as g:profile^42^, PANTHER^43^, TopFun^31^, InterMine^44^, GoEast^45^, and GeneTrail2^46^ provide up-to-date knowledgebases, a significant portion of portals (60%) relied on knowledgebases older than one year (Supplementary Data 1). Due to the inclusion of over 40 unique data sources in Metascape, a fully automated synchronization pipeline becomes unstable due to changes in the underlying resources. This motivated us to develop a workflow that combines an automated robot crawling phase with a supervised human review phase (Fig. 4). This data-engineering solution has demonstrated success through our monthly data refresh cycles, providing a viable data synchronization model for complex portals facing similar challenges.

In addition to regular database updates, gene annotation and membership search are two features that are not readily addressed by existing portals (Supplementary Data 1). Although the term gene annotation is occasionally used by existing portals as a synonym for enrichment analysis^44,47,48^, here we consider gene annotation as a method to extract a diverse set of metadata, such as identifying extracellular proteins or associated chemical probes, for hundreds to thousands of gene candidates at once. Membership search is a closely related yet complementary service that dynamically highlights genes known to be involved in specific biology, such as host-pathogen interactions. Typically, retrieving these metadata requires querying specific portals, often one gene at a time, and compiling outputs into a centralized format for analysis. Metascape enables the batch extraction of contents from over 40 databases in an extremely efficient manner (Supplementary Figures 3–4).

Analyzing multiple gene lists simultaneously through robust meta-analysis is another unmet need in the current OMICs data analysis landscape (Supplementary Data 1). Evaluated portals provide no, or only limited, support for this activity. For example, GoEast^45^ and agriGO^49^ provide some meta-analysis functions using a two-step process, where users analyze gene lists individually first, then reupload their enrichment results for cross comparison. However, GoEast analyzes a maximum of three lists and agriGO only supports organisms of agricultural relevance. GeneCodis^50^ supports a maximum of two lists. GoMiner^51^ can compute enrichment terms for multiple lists in parallel, however, the separate outputs are not further integrated and cross compared. g:Cocoa, a component in g:profile, compiles a tabular output conceptually similar to Metascape’s enrichment heatmap. However, it currently lacks an enrichment clustering feature and does not support interactome meta-analysis (Fig. 2b). Metascape’s architecture is designed to support a seamless user experience towards the analysis and visualization of an arbitrary number of gene lists, only limited by available computing time and memory space.

The integration of Circos plots overcomes the limitation of Venn diagrams to graphically visualize the overlaps among many lists (Supplementary Figure 11), and enrichment heatmaps (Fig. 3a) are applicable to the number of lists encountered in practice (Fig. 3a). Metascape also builds on previous implementations of enrichment network maps^52^ by depicting enriched terms as pie charts, which more readily indicate common and selective pathways enriched across multiple gene lists (Fig. 3b).

While interactome analysis is implemented in Babelomics^53^, Intermine^44^, and ConsensusPathDB^54^, Metascape’s interactome analysis is distinguished by two notable features. First, in addition to the BioGrid knowledgebase, Metascape integrates the more recent human interactome datasets InWeb_IM^55^ and OmniPath^56^, resulting in a tripling of human interactome coverage (Supplementary Figure 13). Second, Metascape automatically extracts protein complexes and annotates their respective functions (Fig. 2h, Supplementary Figure 12). Compared to existing visualization approaches (Fig. 2f), Metascape is designed to highlight dense interactome neighborhoods and facilitate the interpretation of interactome data in a biological context; it further integrates with Cytoscape^29^ to leverage its rich set of visualization and interactive features.

Critically, Metascape was designed to provide more interpretable and actionable results for experimentalists. The redundancy among ontology terms presents a significant obstacle in result interpretation. The primary strategy adopted by existing portals, as seen in g:Profiler^42^, WebGestalt^8^, Babelomics^53,^ and others, is to utilize directed acyclic graph (DAG) or tree structures embedded within ontologies such as GO^16^ (examples in Fig. 2a, b). WebGestalt provides an option only using a subset such as GOSlim^16^ to reduce redundancy. However, these strategies are not applicable to reduce redundancies across different ontology sources. Metascape and DAVID^6^, by contrast, adopt a dynamic clustering solution that avoids the above-mentioned shortcomings. Metascape further simplifies the result presentation via bar graph (Fig. 2d) and heatmap (Fig. 3a) visualizations, compared to DAVID’s tabular presentation (Fig. 2c). Although *p*-value is used as the default representation metric for ranking enriched terms, it nevertheless has its limitations. Users may need to consider using alternative metrics, such as the multi-test corrected *q*-value or the biological context-sensitive enrichment factor. All popular enrichment metrics, including detailed gene counts, are computed and made available for offline analyses. Metascape further enhances the interpretability of analysis results through advanced graphical and spreadsheet outputs, a concise PowerPoint presentation (Supplementary Figure 7), and a journal-style web-based Analysis Report containing additional method descriptions and references (Supplementary Figure 7). Offline analysis is facilitated by a Zip export containing third-party compatible data files, such as the Cytoscape^29^ and JTreeView^57^ formats.

In conclusion, Metascape has been designed for experimentalists (bench biologists) to apply powerful computational analysis pipelines to analyze and interpret large-scale datasets. To ensure that its content is kept current, a new knowledgebase synchronization pipeline was engineered. To address gaps in current analysis tools, Metascape introduces a workflow integrating gene annotation, membership analysis, and multi-gene-list meta-analysis capabilities. Its rich set of analysis tools are accessible through a convenient one-click Express Analysis interface and results are communicated via an article-like analysis report. We expect that the wider adoption of Metascape will significantly enhance biological interpretation of OMICs studies by enabling experimentalists to directly analyze their data to identify novel therapeutic targets, mechanisms of action, or molecular insight into disease. Metascape is an open-access resource available at http://metascape.org.

## Methods

### Architecture and data source

Metascape is a web resource compatible with all major browsers. The backend knowledgebase is a relational database managed by MySQL. The front-end user interface is implemented by Angular JavaScript framework to provide a rich interactive user experience. The server-side computational logic is mostly implemented in Python and leverages several packages provided by the community (https://pypi.org). Each analysis request is associated with a randomly-generated unique session ID (Supplementary Figure 2), which can be used to access analysis results interactively through the web or to download all result data in a Zip package for offline use, before the data is removed from the server (Supplementary Figure 7).

The rich analyses of Metascape sit on top of over 40 bioinformatics knowledgebases maintained by the scientific community (Supplementary Figure 8, Supplementary Data 2). A data synchronization pipeline, as described in Fig. 4, is applied monthly to process data into an Entrez Gene ID-centric relational data format. Metascape currently supports ten organisms: *H. sapiens*, *M. musculus*, *R. norvegicus*, *D. rerio*, *D. melanogaster*, *C. elegans*, *S. cerevisiae*, *A. thaliana*, *S. Pombe*, and *P. falciparum*.

### Functional enrichment analysis

Metascape utilizes the well-adopted hypergeometric test^58^ and Benjamini-Hochberg *p*-value correction algorithm^59^ to identify all ontology terms that contain a statistically greater number of genes in common with an input list than expected by chance. By default, Metascape pathway enrichment analysis makes use of Gene Ontology^16^, KEGG^17^, Reactome^18^, MSigDB^19^, etc. Distinguishing it from many existing portals, Metascape automatically clusters enriched terms into non-redundant groups, where it implements similar logic as found in DAVID^6^. Briefly, pairwise similarities between any two enriched terms are computed based on a Kappa-test score^28^. The similarity matrix is then hierarchically clustered and a 0.3 similarity threshold is applied to trim the resultant tree into separate clusters. Metascape chooses the most significant (lowest *p*-value) term within each cluster (Supplementary Data 4) to represent the cluster in bar graph and heatmap representations. The analysis provides other popular enrichment metrics in addition to *p*-values.

### Interactome analysis

Metascape utilizes physical protein–protein interactions captured in BioGrid^24^ as the main data source. In addition, it integrates more recent human interactome datasets including InWeb_IM^55^ and OmniPath^56^ to provide additional interactome coverage (Supplementary Figure 13). Given a list of proteins, it first automatically extracts a protein interaction network formed by these candidates. Then for each connected network component, it iteratively applies the MCODE algorithm^33^, with modifications for performance improvements, to identify densely-connected complexes. For each complex, it further applies function enrichment analysis and uses the top three enriched terms for the annotation of its biological roles (Fig. 2h, Supplementary Figure 12). All network visualizations are generated with Cytoscape^29^ and exported in multiple formats.

### Datasets

Previously published host factors that regulate influenza replication were used as example gene lists in this study. The 121 host factors identified by Brass et al.^13^ were used as a single gene list input for all portals studied in Supplementary Data 1 and Fig. 2. To demonstrate meta-analysis capabilities, three influenza gene lists were used as the input, including 121 host factors from Brass et al.^13^, 168 from Karlas et al.^14^, and 294 from Konig et al.^15^. All gene lists were downloaded from the IAV database^9^.

### User interface

Supplementary Figures 1–7 describe the Metascape user interface step-by-step for gene-list analyses using the above-mentioned datasets. Screenshots specific to the single gene list analysis are outlined in orange; those specific to the multiple gene list meta-analysis are outlined in green; common screenshot components are outlined in gray. During the Express Analysis, the submission of gene list(s) (Supplementary Figure 1) directly leads to an analysis report (Supplementary Figure 7) without the need to step through the intermediate operations (Supplementary Figures 2–6). Conversely, the detailed analysis interface (Supplementary Figures 2–6) is only accessible through Custom Analysis.

### Usage statistics

To avoid statistics bias due to new users, who tested and learned Metascape using either example gene lists provided on the portal site or users’ own lists, all analysis sessions associated with an exact same input gene-list group were considered duplicates, regardless of users’ IP address and the time of submission. With such duplicates being removed first, statistics on web site analysis pattern were compiled based on over 70,000 most recent sessions by the time of writing. The relative percentage usage of Metascape visualizations and spreadsheets are derived based on a total of 241 publications (2016-current), implying a maximum standard deviation of 3% in the estimations.

## Supplementary information

Supplementary Information

Description of Additional Supplementary Files

Supplementary Data 1

Supplementary Data 2

Supplementary Data 3

Supplementary Data 4

Supplementary Data 5

## Data Availability

Gene lists referenced in this study are available in Supplementary Data 3.

## Code availability

Custom code used in this study is available from the corresponding authors upon reasonable request.
