# Supplementary material for: Metascape provides a biologist-oriented resource for the analysis of systems-level datasets
Source: Nat Commun. 2019 Apr 3;10:1523. doi: 10.1038/s41467-019-09234-6 (PMC6447622; doi:10.1038/s41467-019-09234-6)
Supplement: Supplementary file 1 — Supplementary Information [file 41467_2019_9234_MOESM1_ESM.pdf]

SUPPLEMENTARY INFORMATION

Metascape provides a biologist-oriented resource for the analysis of systems-level datasets

Zhou *et al.*

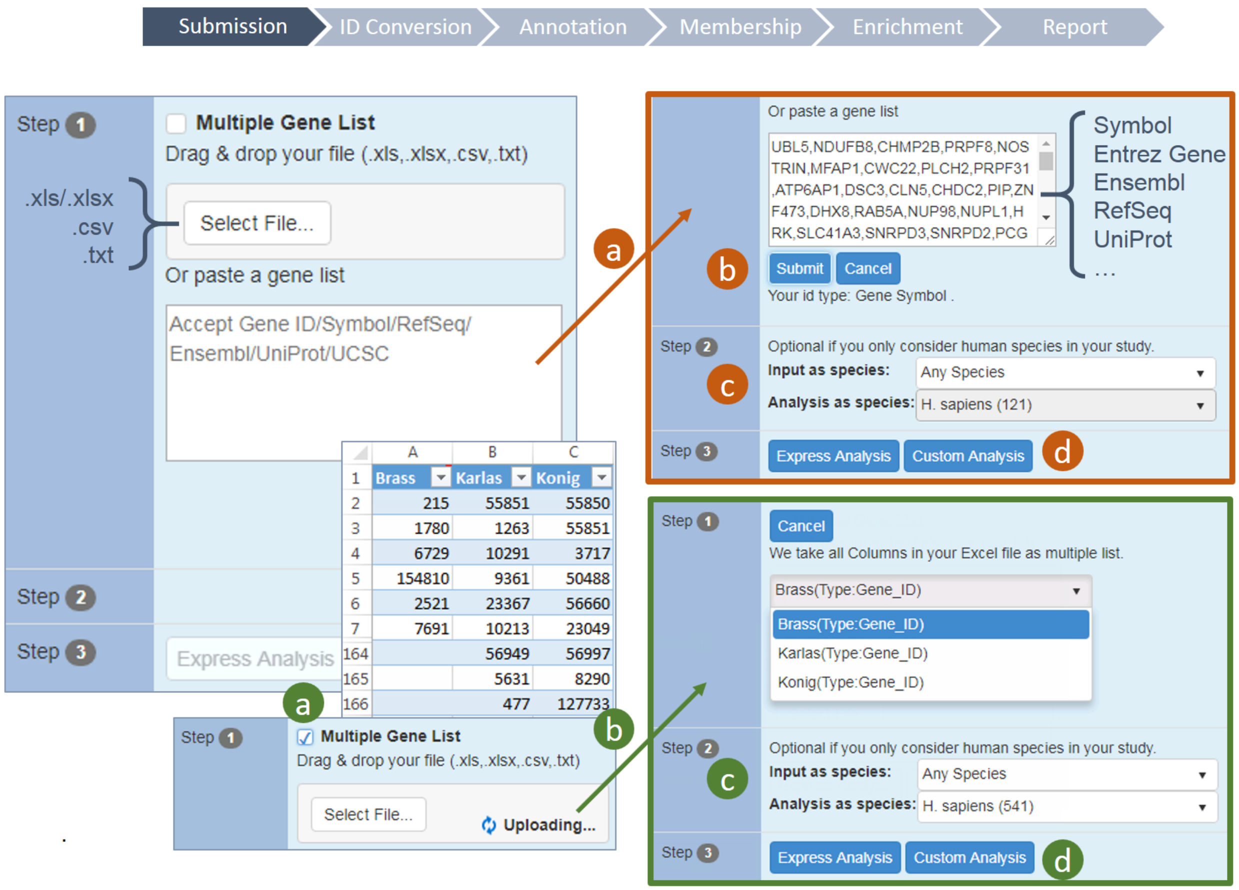

Supplementary Figure 1. Gene list submission user interface. For the single-list submission, (a) users paste gene identifiers (gene symbols in this example) into the text area and (b) submit. (c) Metascape automatically recognizes the input identifier type as Gene Symbol and the default analysis species is set to human with all 121 input gene identifiers recognized. Metascape supports other gene identifier types, such as Entrez Gene ID, Ensembl ID, RefSeq, UniProt ID, etc. (d) Finally, users choose either Express Analysis or Custom Analysis to launch the CAME analysis pipeline. To submit multiple gene lists, (a) users first check the Multiple Gene List box, (b) then drag and drop the file containing input gene lists. Metascape supports multiple formats including .xls/.xlsx, .csv, or .txt (see example submission files on the web site for the exact format requirements). Here, three gene lists are read and a total of 541 human genes are recognized. Similar to the single list case, users can (c) change analysis species and (d) trigger the analysis workflow. The background gene list for enrichment analysis defaults to the whole genome. However, users can optionally provide a custom background gene list named "_BACKGROUND" through the file submission interface, which supports the upload of both single and multiple gene lists. Users who choose Custom Analysis are still able to supply customized background genes at the later enrichment analysis step as well. Users who choose the Express Analysis route wait for the analysis progression bar to reach 100%, then click on an Analysis Report Page button to directly jump to the output page in Supplementary Figure 7. Supplementary Figures 2-6 are for Custom Analysis only.

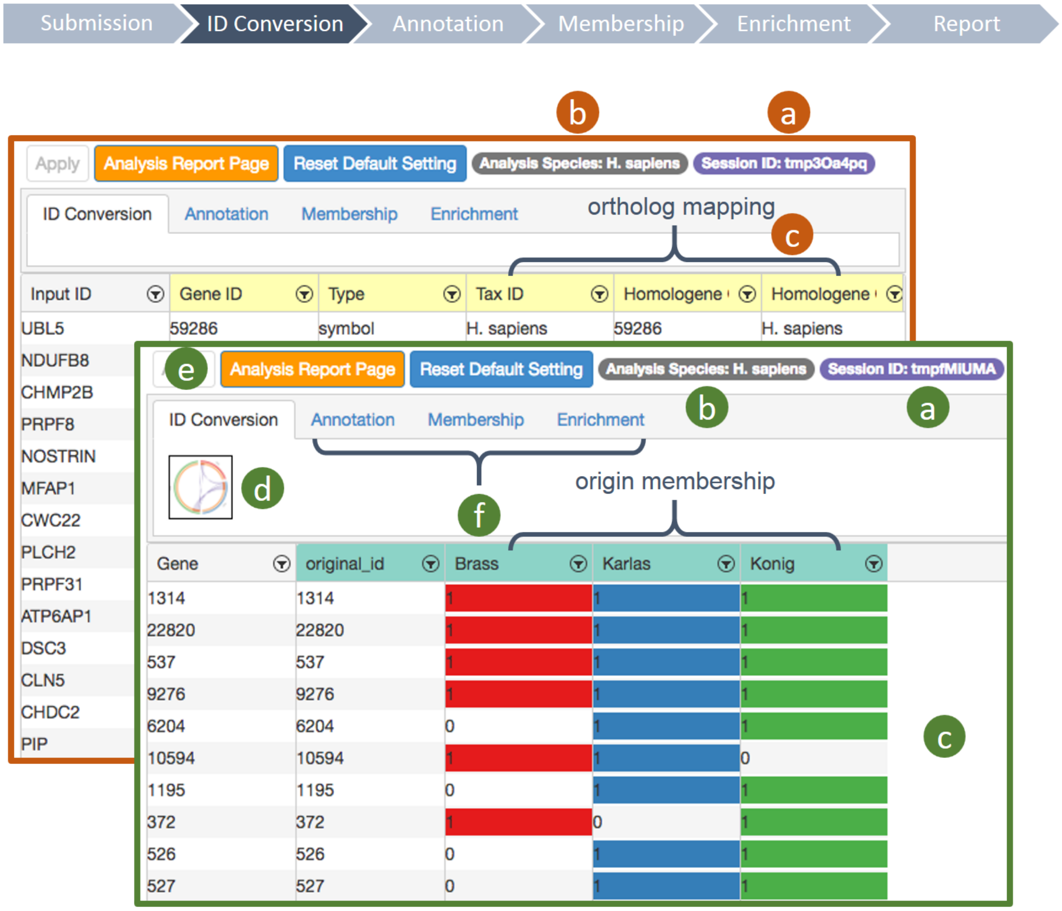

Supplementary Figure 2. Identifier conversion user interface. ID conversion is automatically carried out as the only mandatory CAME analysis step without user interaction. (a) Metascape first assigns a unique random session ID identifying the specific submission. The results of this analysis session can either be accessed online using the session ID for a limited time (currently set to three days) or be downloaded as a Zip archive. The input gene identifiers are mapped onto Entrez Gene IDs, including mapping deprecated Entrez Gene IDs to newer IDs according to the NCBI database. (b) All analyses are carried out using the knowledgebase associated with the specified target analysis species. (c) If the target analysis species differs from the input species, ortholog mapping is automatically applied. The ambiguity of potentially mapping one input gene into multiple orthologs is resolved by choosing the ortholog with the largest number of PubMed publications, i.e., it is assumed the best-studied ortholog is the one likely to yield most biological context in later analyses. Duplications in the resultant gene list are removed. Multi-list analyses share the same steps (a) and (b). (c) In contrast to single-list analysis, the three input gene lists are combined into one combined gene list consisting of all unique input candidates, with the origin of each gene and its source gene list(s) preserved under the corresponding binary membership columns (Supplementary Figure 10, Supplementary Data 4). For the ease of visualization, each gene list is automatically assigned a unique color (red for Brass, blue for Karlas, and green for Konig) throughout the remainder of the session. (d) The gene-level overlap among the three lists is visualized by a Circos plot (Supplementary Figure 11A), where the same color code is used. (e) As the remaining steps of the CAME analyses are optional, the ID conversion results are immediately available through an Analysis Report button. (f) To continue with additional CAME analyses, users can activate the corresponding tabs. As Metascape is designed to support an arbitrary number of gene lists, the user interface is consistent and largely identical between the single-list and multi-list use cases.

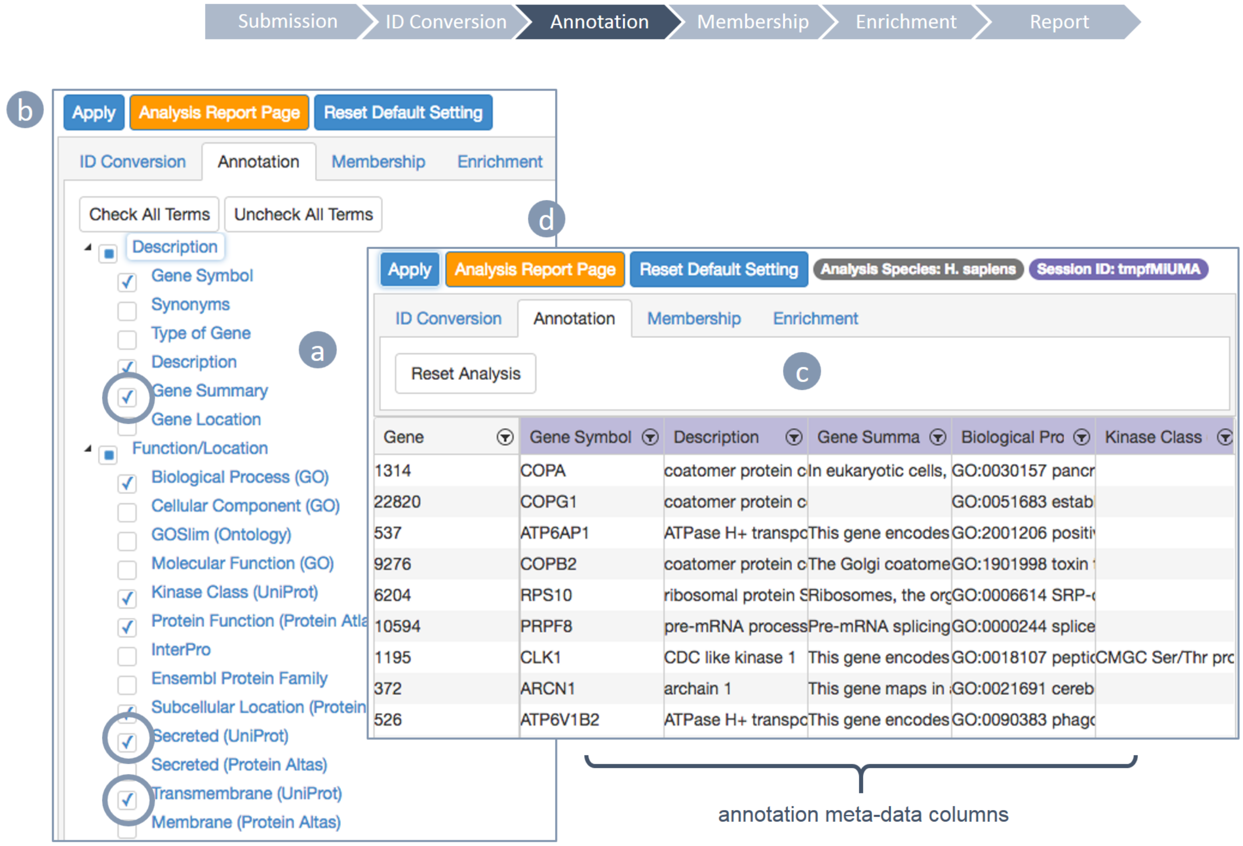

Supplementary Figure 3. Gene annotation user interface. Since the ID conversion analysis generates one combined gene list, the gene annotation interface is identical regardless of the number of supplied gene lists. Metascape enables users to take advantage of a rich metadata set supported by over 40 underlying knowledgebases to provide comprehensive biological context for OMICs data interpretation. (a) Users choose metadata columns from a total list of 47 options covering gene description, gene function and subcellular locations, genotype/phenotype/disease associations, tissue expression, ontology annotation, and other miscellaneous categories. By default, the nine most popular terms are selected in the Express Analysis, including gene symbol and description from NCBI, biological process association from GO, kinase class from UniProt, protein function and subcellular location from Protein Atlas, chemical probes from DrugBank, and canonical pathways and hallmark gene set associations from MSigDB. However, users can overwrite the defaults. The example shown requests the gene summary from NCBI, as well as the secreted and transmembrane gene prediction results from UniProt. (b) Clicking on the Apply button signals Metascape to batch retrieve the requested metadata in seconds and (c) append them to the resultant data sheet as individual metadata columns (Supplementary Data 3). (d) In addition to the online view, the spreadsheet accumulating all analysis results so far can be downloaded using the Analysis Report Page button.

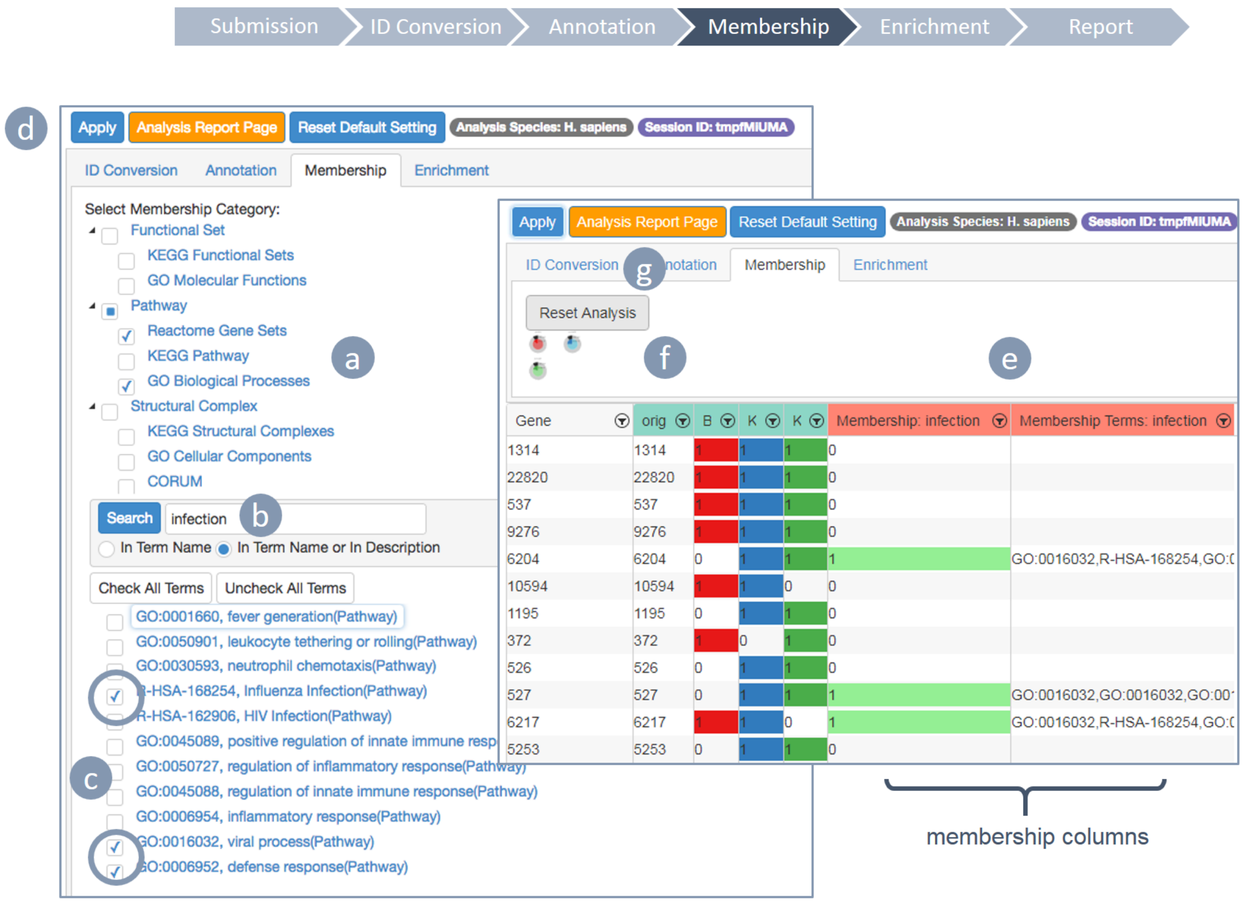

Supplementary Figure 4. Membership search user interface. The example above demonstrates how to mark known influenza-related host factors within the input gene candidates. The interface is identical regardless of the number of gene lists. (a) Users first select the ontology categories to query (Reactome and GO Biological Process in this example), (b) then enter a search term (“infection” in the example). (c) From the returned list of matched terms, three were chosen: “influenza infection” from Reactome, “viral process” and “defense response” from GO. (d) Users then click on the Apply button to identify all candidates associated with these three terms. (e) Two membership data columns are appended to the resultant sheet, containing a binary column marking all known host factors and an annotation column listing the underlying matched terms (Supplementary Data 3). (f) Membership analysis results are always binary, segregating candidate genes into two groups. A Chi-square enrichment analysis is then automatically applied to evaluate whether the list contains an unusually large number of “yes” members. Each membership analysis is therefore associated with one p-value per list and can be visualized by a nested pie chart (Supplementary Figure 9). (g) The Analysis Report Page button can be activated to allow access to all analysis results accumulated through this step.

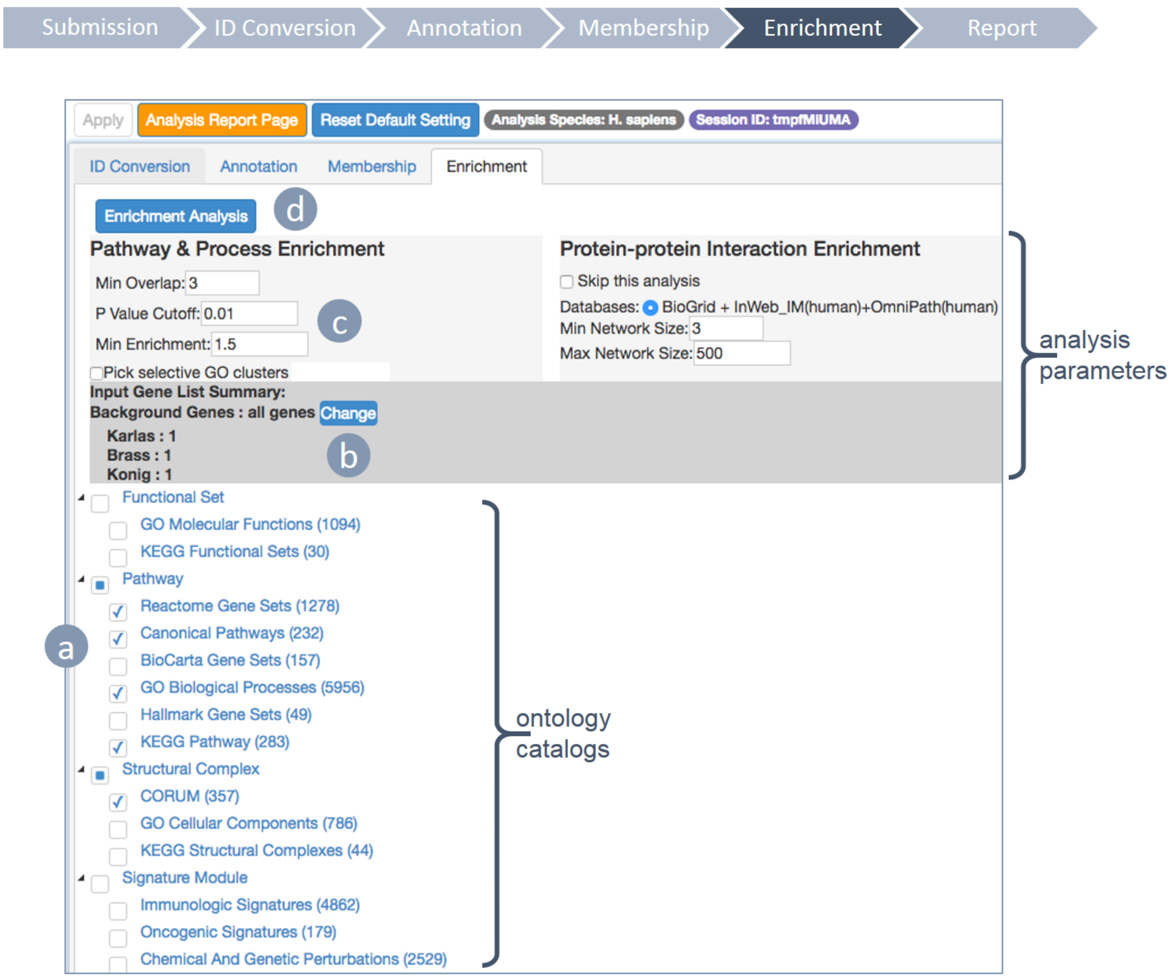

Supplementary Figure 5. Functional enrichment and interactome analysis settings user interface. This interface is identical regardless of the number of gene lists analyzed. (a) Users specify the ontology catalogs that are relevant to the purpose of the study. By default, GO biological process, KEGG pathways, Reactome gene sets, CORUM complexes, and canonical pathways from MSigDB are selected. (b) If a custom background gene list has not yet been provided (Supplementary Figure 1), one can be supplied now using any gene identifiers supported by Metascape. The custom background list is automatically amended to include any foreground candidate genes that happen to be missing in the background pool. (c) Users can also overwrite the default analysis parameters, such as requiring enriched terms to include ≥ 3 candidates, p-value ≤ 0.01, and enrichment factor ≥ 1.5, or limiting interactome analysis to networks containing 3 to 500 candidate proteins. Although Metascape prioritizes results for terms that are commonly shared across gene lists, users can check “Pick selective GO clusters” to adopt a different prioritization algorithm to preferentially identify terms that are selective across gene lists instead. (d) The Enrichment Analysis button triggers the selected analyses and the results are first displayed online as shown in Supplementary Figure 6.

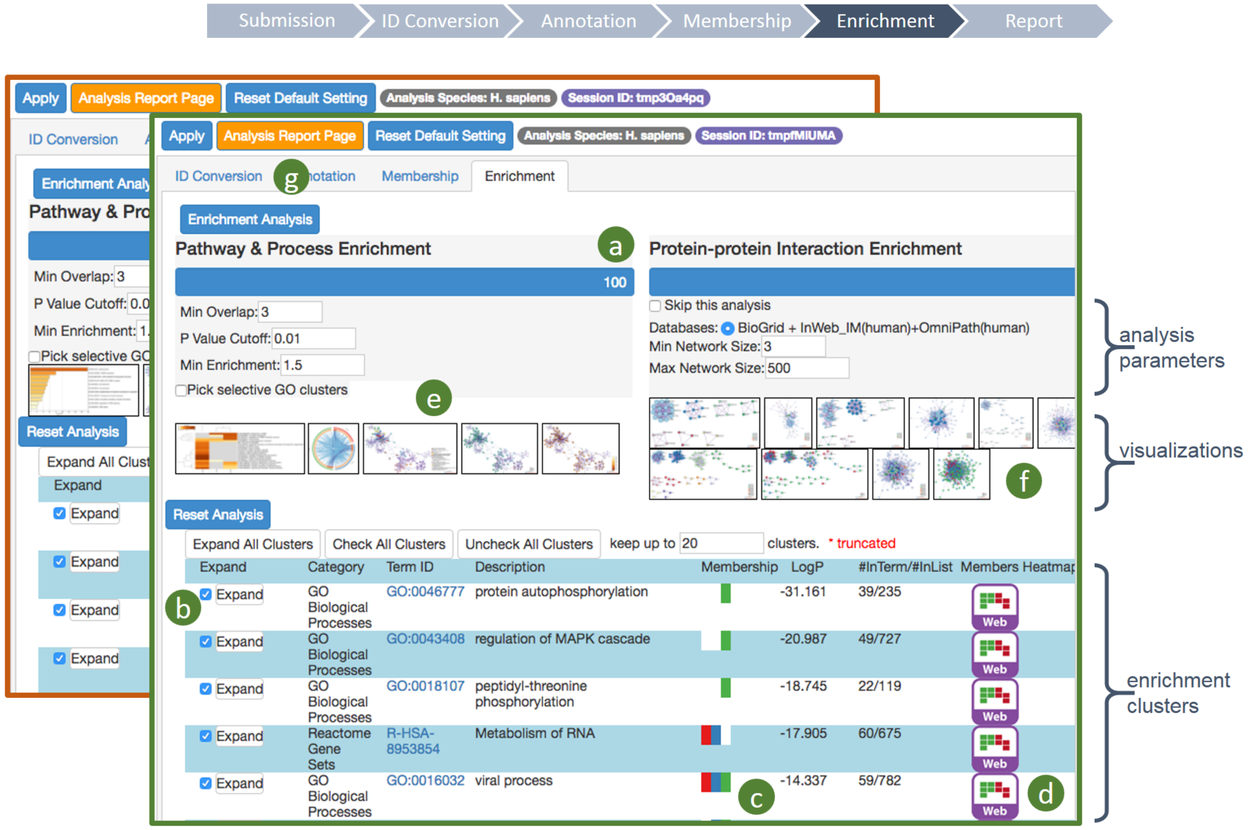

Supplementary Figure 6. Functional enrichment and interactome analysis output user interface. The interface of the single- and multi-gene-list analysis is very similar, therefore, we only describe the meta-analysis snapshot. (a) After the enrichment and interactome analysis progress bars both reach 100%, the results are rendered on the web page. (b) All enriched ontology terms are binned into non-redundant clusters and shown in a tabular format where users can expand clusters to view the underlying terms. The table lists p-values and gene counts in both foreground and background. (c) The Membership column indicates the gene lists where the corresponding cluster was found to be enriched, e.g. “viral process” is enriched for all three lists with red, blue and green color bars shown, while “protein autophosphorylation” has only a green color bar indicating that it is specific to the Konig list. (d) Links to online clustergrams allow interactive review of the associations between gene candidates and enriched terms. (e) Enrichment results are also visualized by a heatmap for the multi-list analysis (Figure 3a) or a bar graph for the single-list analysis, together with enrichment networks colored by p-values or cluster IDs (Figure 2e) or pie charts (Figure 3b). For meta-analysis, a new Circos plot is generated to depict the overlap of the multiple gene lists at the biological function level (Supplementary Figure 11B). (f) The protein networks constructed based on physical interactions among all input protein (gene) candidates, or individual protein (gene) lists are visualized, with MCODE complexes separately rendered as well as highlighted in the global network (Figure 2g-h, Supplementary Figure 12). (g) All analysis results can be viewed and downloaded through Analysis Report Page.

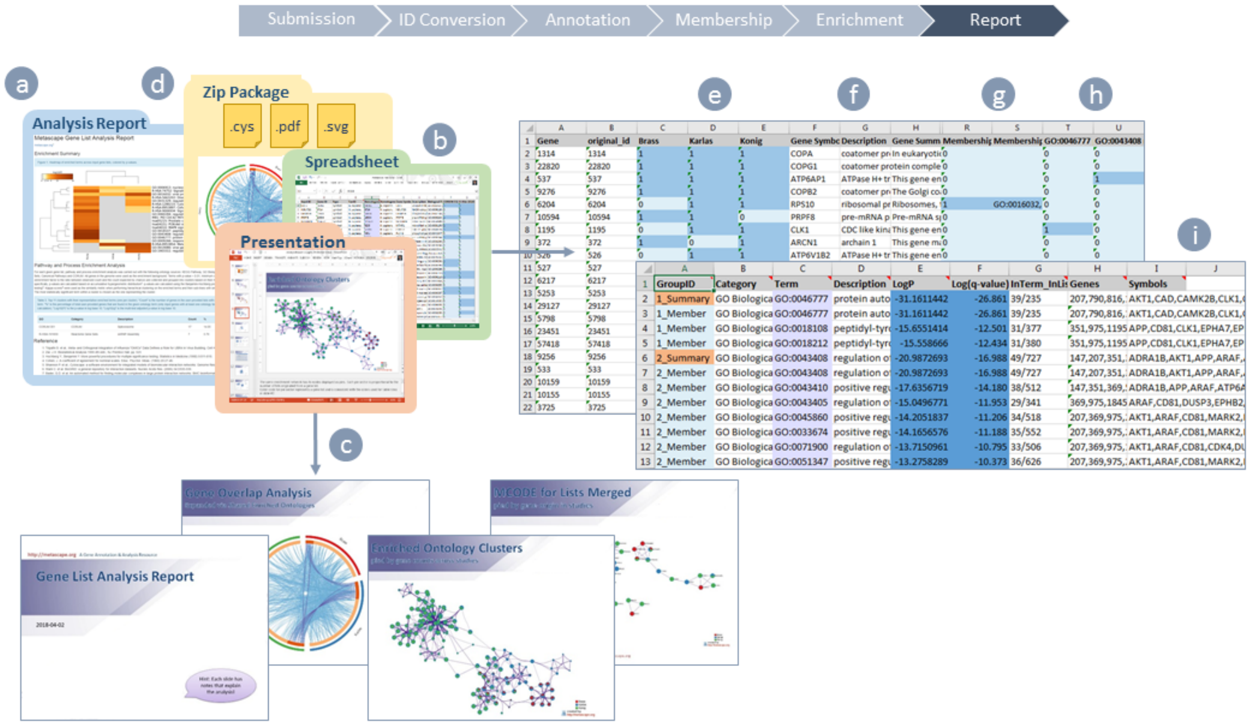

Supplementary Figure 7. Analysis report page user interface. (a) The Analysis Report Page is an article-style web report that summarizes the input gene lists, enrichment bar graph or heatmap, enrichment table, enrichment networks, interactome networks, together with detailed underlying materials and methods, and completed with corresponding reference information. The top of the report page contains links to other report formats, including (b) an Excel workbook, (c) a PowerPoint presentation and (d) an all-in-one Zip package. (b) The Excel file contains two data sheets. The first, a gene annotation sheet, is essentially an evidence matrix including (e) membership columns preserving the origin of each gene candidate, (f) metadata columns extracted by gene annotation analysis, (g) membership search columns flagging candidates associated with terms resulting from a keyword search, and (h) enrichment membership columns capturing the association between genes and enriched pathway clusters (Supplementary Data 3). (i) The enrichment summary sheet includes all key statistics and annotation data for the enriched pathway clusters and underlying terms (Supplementary Data 4). (c) The PowerPoint presentation contains key summary data and visualizations, as well as detailed notes explaining the underlying analysis algorithms. (d) All these files, including the web report itself, are downloadable as a Zip package for offline sharing and retention. The Zip package also contains result files in relevant third-party formats, which facilitate further data manipulation using sophisticated tools dedicated to specific analyses. For example, all networks are available as .xgmml and .cys formats supported by Cytoscape[52], and heatmap files are provided for JTreeView[53]. Besides supporting popular graphic formats, such as .png, for online viewing, all visualizations have also been made available in publication-ready formats, such as .pdf and/or .svg.

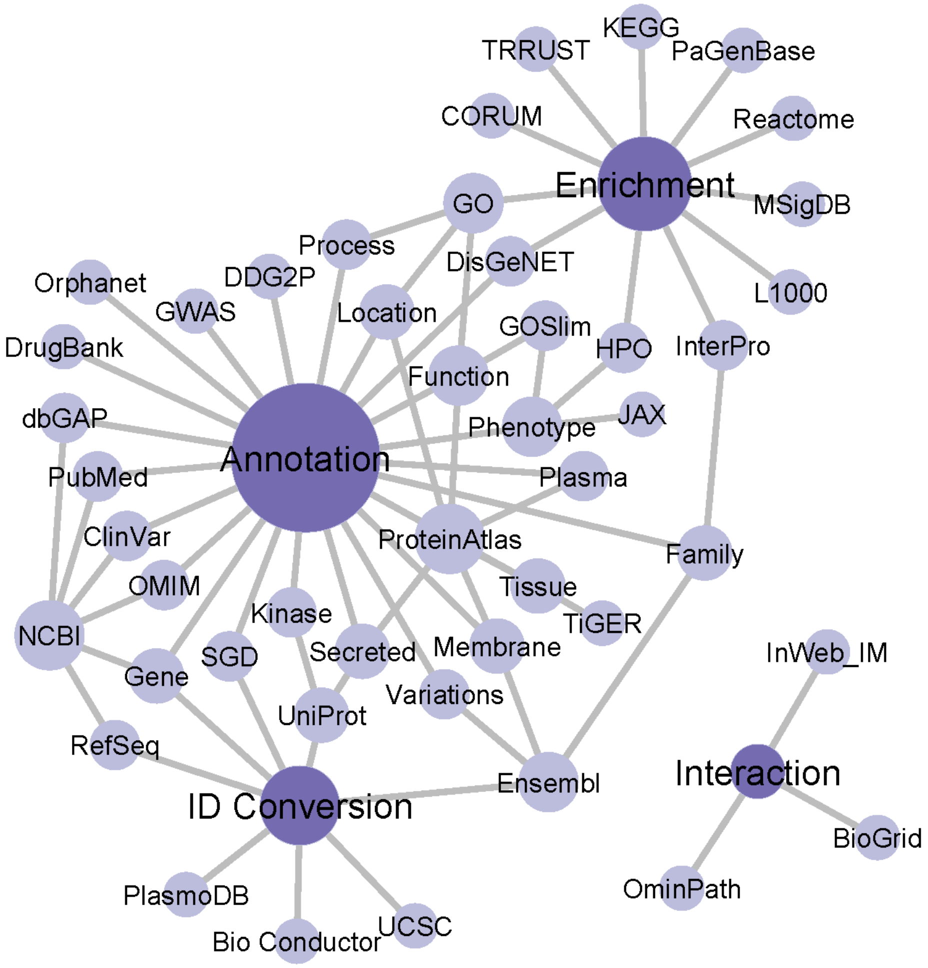

Supplementary Figure 8. Over 40 data sources are integrated in Metascape to support its CAME analysis workflow (also see Supplementary Data 2).

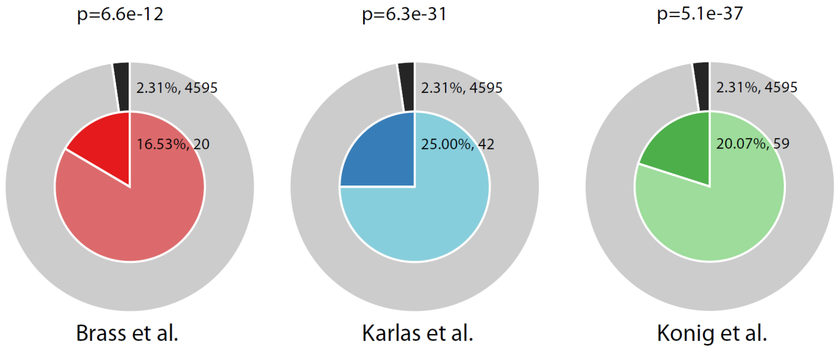

Supplementary Figure 9. Pie chart visualization of the enrichment of membership search terms related to “infection” in the three influenza host factor gene lists. Genes associated with the following three matched terms were flagged: R-HAS-168254 “influenza infection” from Reactome, GO:0016032 “viral process” and GO:0006952 “defense response” from GO biological process. The inner pie represents the portion of infection-related gene candidates within the three individual gene lists; the outer ring depicts the portion of similar genes within the whole genome background. All three lists show statistically significant enrichment of known pathogen-related host factors, where the inner dark pies are larger than their corresponding outer black pies. The same visualization can also be used to present functional enrichment results[54].

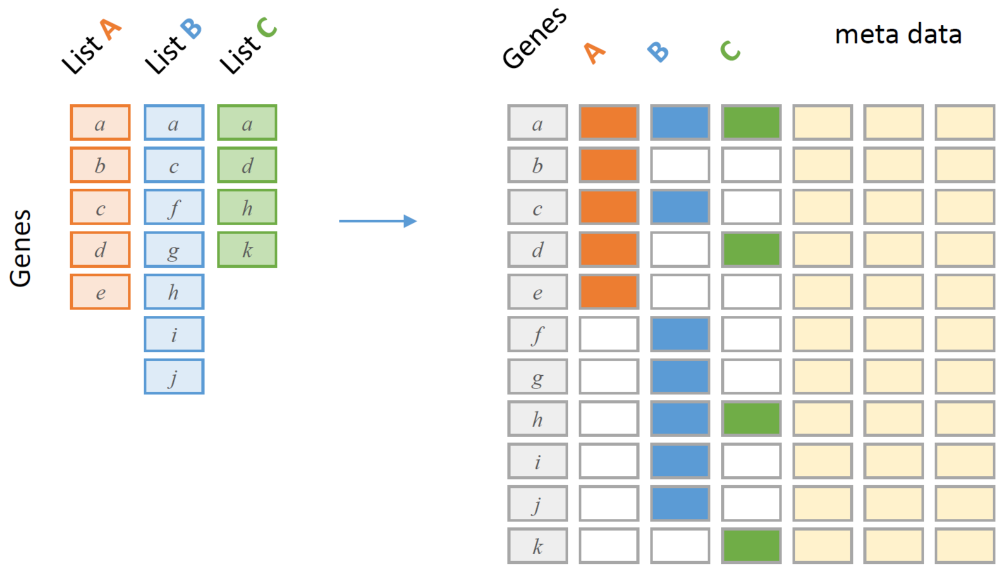

Supplementary Figure 10. Schematic illustration of the meta-analysis result representation strategy. The input *n* gene lists (left) are pivoted into an evidence matrix (right), where rows correspond to unique genes and the first *n* binary membership columns indicates whether a gene originated from a specific input list. Gene *a* here, for instance, is associated with all three lists. Metadata extracted by gene annotation and membership search analyses are then appended as additional columns afterwards.

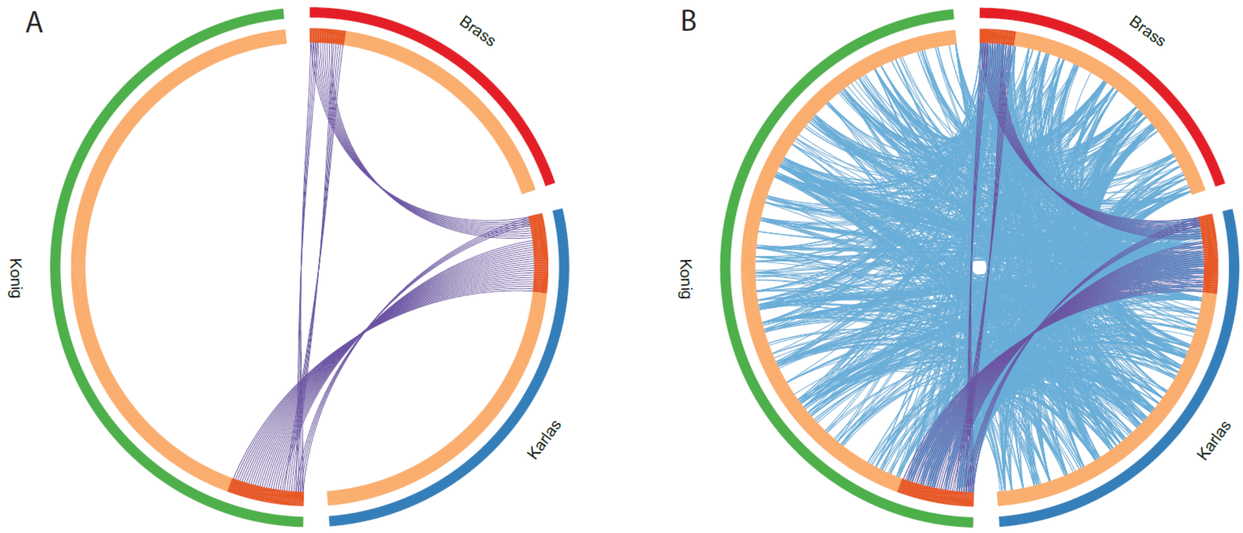

Supplementary Figure 11. Circos plot visualization of the overlaps among gene lists. (A) Each candidate gene is assigned to one spot on the arc of the corresponding gene list(s). Genes shared among multiple lists are linked through purple curves. (B) Blue curves link those candidate genes that have different identities but share an enriched pathway/process, i.e. they represent the functional overlaps among gene lists.

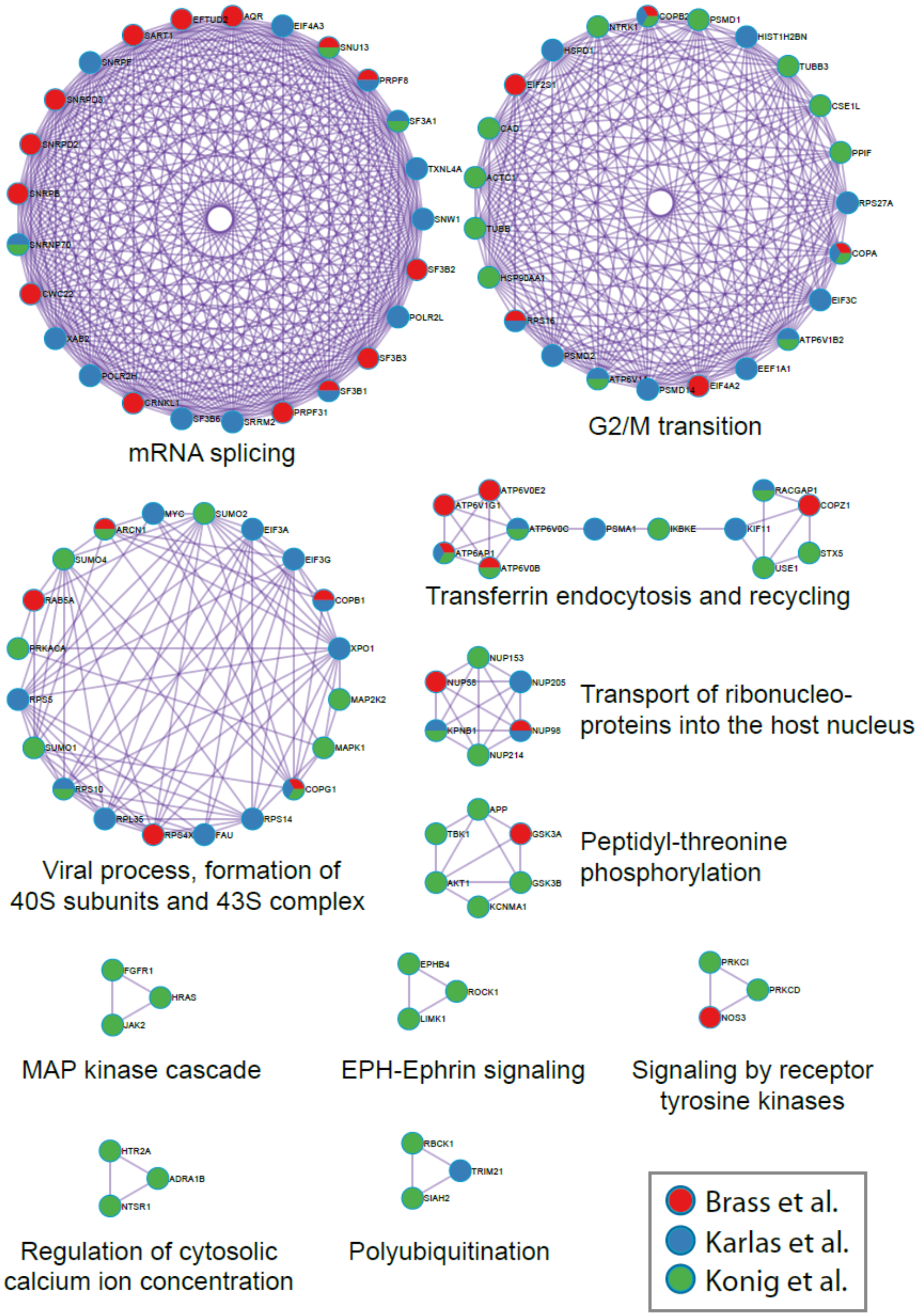

Supplementary Figure 12. Eleven MCODE complexes extracted from the interactome network formed by 541 unique proteins based on the combination of the Brass, Karlas, and Konig gene lists. Each node represents a protein in pie chart style, where each color encodes its origin. Complexes involved in "G2/M transition", "viral process, formation of 40S subunits and 43S complex", "transferrin endocytosis and recycling", and "transport of ribonucleoproteins into the host nucleus" are shared among all three lists. The "mRNA splicing" complex is predominately shared between the Brass and Karlas lists. The remaining complexes are mostly specific to the Konig list. The results are consistent with the functional enrichment analysis results in Figure 3a.

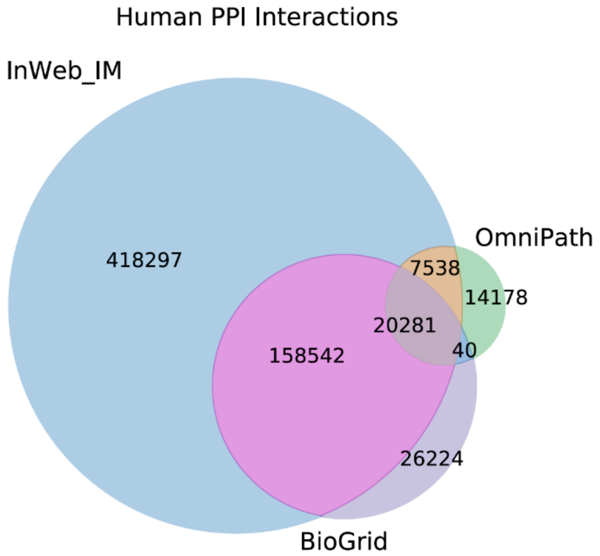

Supplementary Figure 13. The overlap analysis of human interactomes based on different sources. In total, InWeb_IM[37] and OmniPath[38] contain about twice more physical interactions not captured by BioGrid[36].

Supplementary References

1. Alonso R, *et al.* Babelomics 5.0: functional interpretation for new generations of genomic data. *Nucleic acids research* **43**, W117-121 (2015).
2. Huang da W, Sherman BT, Lempicki RA. Systematic and integrative analysis of large gene lists using DAVID bioinformatics resources. *Nature protocols* **4**, 44-57 (2009).
3. Kuleshov MV, *et al.* Enrichr: a comprehensive gene set enrichment analysis web server 2016 update. *Nucleic acids research* **44**, W90-97 (2016).
4. Reimand J, *et al.* g:Profiler-a web server for functional interpretation of gene lists (2016 update). *Nucleic acids research* **44**, W83-89 (2016).
5. Wang J, Vasaikar S, Shi Z, Greer M, Zhang B. WebGestalt 2017: a more comprehensive, powerful, flexible and interactive gene set enrichment analysis toolkit. *Nucleic acids research* **45**, W130-W137 (2017).
6. Mi H, *et al.* PANTHER version 11: expanded annotation data from Gene Ontology and Reactome pathways, and data analysis tool enhancements. *Nucleic acids research* **45**, D183-D189 (2017).
7. Chen J, Bardes EE, Aronow BJ, Jegga AG. ToppGene Suite for gene list enrichment analysis and candidate gene prioritization. *Nucleic acids research* **37**, W305-311 (2009).
8. Kalderimis A, *et al.* InterMine: extensive web services for modern biology. *Nucleic acids research* **42**, W468-472 (2014).
9. Zheng Q, Wang XJ. GOEAST: a web-based software toolkit for Gene Ontology enrichment analysis. *Nucleic acids research* **36**, W358-363 (2008).
10. Castillo-Davis CI, Hartl DL. GeneMerge--post-genomic analysis, data mining, and hypothesis testing. *Bioinformatics (Oxford, England)* **19**, 891-892 (2003).
11. Eden E, Navon R, Steinfeld I, Lipson D, Yakhini Z. GOrilla: a tool for discovery and visualization of enriched GO terms in ranked gene lists. *BMC bioinformatics* **10**, 48 (2009).
12. Herwig R, Hardt C, Lienhard M, Kamburov A. Analyzing and interpreting genome data at the network level with ConsensusPathDB. *Nature protocols* **11**, 1889-1907 (2016).
13. McLean CY, *et al.* GREAT improves functional interpretation of cis-regulatory regions. *Nature biotechnology* **28**, 495-501 (2010).
14. Berriz GF, King OD, Bryant B, Sander C, Roth FP. Characterizing gene sets with FuncAssociate. *Bioinformatics (Oxford, England)* **19**, 2502-2504 (2003).
15. Kang H, *et al.* gsGator: an integrated web platform for cross-species gene set analysis. *BMC bioinformatics* **15**, 13 (2014).
16. Carmona-Saez P, Chagoyen M, Tirado F, Carazo JM, Pascual-Montano A. GENECODIS: a web-based tool for finding significant concurrent annotations in gene lists. *Genome biology* **8**, R3 (2007).
17. Zeeberg BR, *et al.* GoMiner: a resource for biological interpretation of genomic and proteomic data. *Genome biology* **4**, R28 (2003).
18. Stockel D, *et al.* Multi-omics enrichment analysis using the GeneTrail2 web service. *Bioinformatics (Oxford, England)* **32**, 1502-1508 (2016).

19. Tian T, *et al.* agriGO v2.0: a GO analysis toolkit for the agricultural community, 2017 update. *Nucleic acids research* **45**, W122-W129 (2017).

20. Rho K, *et al.* GARNET--gene set analysis with exploration of annotation relations. *BMC bioinformatics* **12 Suppl 1**, S25 (2011).

21. Sartor MA, *et al.* ConceptGen: a gene set enrichment and gene set relation mapping tool. *Bioinformatics (Oxford, England)* **26**, 456-463 (2010).

22. Martin D, *et al.* GOToolBox: functional analysis of gene datasets based on Gene Ontology. *Genome biology* **5**, R101 (2004).

23. Newman JC, Weiner AM. L2L: a simple tool for discovering the hidden significance in microarray expression data. *Genome biology* **6**, R81 (2005).

24. Zhong S, *et al.* GoSurfer: a graphical interactive tool for comparative analysis of large gene sets in Gene Ontology space. *Applied bioinformatics* **3**, 261-264 (2004).

25. Beissbarth T, Speed TP. GOstat: find statistically overrepresented Gene Ontologies within a group of genes. *Bioinformatics (Oxford, England)* **20**, 1464-1465 (2004).

26. Maglott D, Ostell J, Pruitt KD, Tatusova T. Entrez Gene: gene-centered information at NCBI. *Nucleic acids research* **39**, D52-57 (2011).

27. Zerbino DR, *et al.* Ensembl 2018. *Nucleic acids research* **46**, D754-D761 (2018).

28. Kent WJ, *et al.* The human genome browser at UCSC. *Genome Res* **12**, 996-1006 (2002).

29. Ashburner M, *et al.* Gene ontology: tool for the unification of biology. The Gene Ontology Consortium. *Nature genetics* **25**, 25-29 (2000).

30. Kanehisa M, Goto S. KEGG: kyoto encyclopedia of genes and genomes. *Nucleic acids research* **28**, 27-30 (2000).

31. Subramanian A, *et al.* Gene set enrichment analysis: a knowledge-based approach for interpreting genome-wide expression profiles. *Proceedings of the National Academy of Sciences of the United States of America* **102**, 15545-15550 (2005).

32. Subramanian A, *et al.* A Next Generation Connectivity Map: L1000 Platform and the First 1,000,000 Profiles. *Cell* **171**, 1437-1452 e1417 (2017).

33. Han H, *et al.* TRRUST v2: an expanded reference database of human and mouse transcriptional regulatory interactions. *Nucleic acids research* **46**, D380-D386 (2018).

34. Pinero J, *et al.* DisGeNET: a comprehensive platform integrating information on human disease-associated genes and variants. *Nucleic acids research* **45**, D833-D839 (2017).

35. Ruepp A, *et al.* CORUM: the comprehensive resource of mammalian protein complexes--2009. *Nucleic acids research* **38**, D497-501 (2010).

36. Chatr-Aryamontri A, *et al.* The BioGRID interaction database: 2017 update. *Nucleic acids research* **45**, D369-D379 (2017).

37. Li T, *et al.* A scored human protein-protein interaction network to catalyze genomic interpretation. *Nature methods* **14**, 61-64 (2017).

38. Turei D, Korcsmaros T, Saez-Rodriguez J. OmniPath: guidelines and gateway for literature-curated signaling pathway resources. *Nature methods* **13**, 966-967 (2016).

39. Uhlen M, *et al.* Proteomics. Tissue-based map of the human proteome. *Science* **347**, 1260419 (2015).

40. MacArthur J*, et al.* The new NHGRI-EBI Catalog of published genome-wide association studies (GWAS Catalog). *Nucleic acids research* **45**, D896-D901 (2017).

41. Finn RD*, et al.* InterPro in 2017-beyond protein family and domain annotations. *Nucleic acids research* **45**, D190-D199 (2017).

42. Consortium TU. UniProt: the universal protein knowledgebase. *Nucleic acids research* **46**, 2699 (2018).

43. Wishart DS*, et al.* DrugBank 5.0: a major update to the DrugBank database for 2018. *Nucleic acids research* **46**, D1074-D1082 (2018).

44. Blake JA*, et al.* Mouse Genome Database (MGD)-2017: community knowledge resource for the laboratory mouse. *Nucleic acids research* **45**, D723-D729 (2017).

45. Kohler S*, et al.* The Human Phenotype Ontology in 2017. *Nucleic acids research* **45**, D865-D876 (2017).

46. Cherry JM*, et al.* Saccharomyces Genome Database: the genomics resource of budding yeast. *Nucleic acids research* **40**, D700-705 (2012).

47. Aurrecoechea C*, et al.* PlasmoDB: a functional genomic database for malaria parasites. *Nucleic acids research* **37**, D539-543 (2009).

48. *Orphanet: an online database of rare diseases and orphan drugs.* <http://www.orpha.net> (1997).

49. Liu X, Yu X, Zack DJ, Zhu H, Qian J. TiGER: a database for tissue-specific gene expression and regulation. *BMC bioinformatics* **9**, 271 (2008).

50. Huber W*, et al.* Orchestrating high-throughput genomic analysis with Bioconductor. *Nature methods* **12**, 115-121 (2015).

51. Pan JB*, et al.* PaGenBase: a pattern gene database for the global and dynamic understanding of gene function. *PLoS One* **8**, e80747 (2013).

52. Shannon P*, et al.* Cytoscape: a software environment for integrated models of biomolecular interaction networks. *Genome Res* **13**, 2498-2504 (2003).

53. Saldanha AJ. Java Treeview--extensible visualization of microarray data. *Bioinformatics (Oxford, England)* **20**, 3246-3248 (2004).

54. Lotan A. *et al.* Differential effects of chronic stress in young-adult and old female mice: cognitive-behavioral manifestations and neurobiological correlates. *Mol Psychiatry* **23**, 1432-1445 (2018).
