# Supplementary material for: Metascape provides a biologist-oriented resource for the analysis of systems-level datasets
Source: Nat Commun. 2019 Apr 3;10:1523. doi: 10.1038/s41467-019-09234-6 (PMC6447622; doi:10.1038/s41467-019-09234-6)
Supplement: Supplementary file 2 — Description of Additional Supplementary Files [file 41467_2019_9234_MOESM2_ESM.docx]

**Description of Additional Supplementary Files**

File Name: Supplementary Data 1

Description: The study of gene list analysis portals provides a high-level overview of the current landscape of online OMICs data analysis tools. Functional enrichment analysis is the core feature of most portals. There are significant opportunities to enhance support for bench biologists, particularly by adding gene annotation and membership search, as well as maintaining regularly updated databases. Analysis of multiple gene lists is another important feature with limited options currently available.

File Name: Supplementary Data 2

Description: The set of diverse knowledgebases that provide current biological understanding of the genome; these data sources are integrated and applied to support the CAME analysis workflow within Metascape (Supplementary Figure 8).

File Name: Supplementary Data 3

Description: Default gene annotation spreadsheet output by Metascape as the result of Express Analysis on the three influenza host factor lists. Columns B-D indicate the origin of each gene from the submitted gene lists. E-R are meta-data columns containing gene annotation information providing biological context, including their functions, subcellular locations, and available chemical probes, etc. Columns S-T host the results of membership search with the keyword “infection”. Columns U-AN are enrichment membership columns, indicating the association of each gene with 20 enriched pathway clusters. Column AO is the total number of gene lists that a gene candidate was found to be a hit in, which can be used to rank gene candidates similar to a Venn diagram analysis.

File Name: Supplementary Data 4

Description: Default enrichment summary spreadsheet output by Metascape as the result of Express Analysis on the three example gene lists. Each cluster starts with the representative term (which has the lowest p-value within the cluster), followed by all other cluster members. Columns B-D contain meta-data describing the terms. Columns E and F contain the enrichment p-values and multi-test-corrected q-values. Columns H & I enumerate the genes found in each enriched term.

File Name: Supplementary Data 5

Description: Statistics of how various Metascape visualizations and spreadsheets have been applied to support research in 150 publications.
